# Effectiveness and reach of a directed-population approach to improving dental health and reducing inequalities: a cross sectional study

**DOI:** 10.1186/1471-2458-13-778

**Published:** 2013-08-27

**Authors:** Lynn Brewster, Andrea Sherriff, Lorna Macpherson

**Affiliations:** 1 Glasgow Dental Hospital & School, 378 Sauchiehall Street, Glasgow G2 3JZ, Scotland

**Keywords:** Targeted intervention, Directed-population, Proportionate universalism, Increased-risk, Inequalities, Deprivation, Caries

## Abstract

**Background:**

Childsmile School adopts a directed-population approach to target fluoride varnish applications to 20% of the primary one (P1) population in priority schools selected on the basis of the proportion of enrolled children considered to be at increased-risk of developing dental caries. The study sought to compare the effectiveness of four different methods for identifying individuals most in need when a directed-population approach is taken.

**Methods:**

The 2008 Basic National Dental Inspection Programme (BNDIP) cross-sectional P1 Scottish epidemiological survey dataset was used to model four methods and test three definitions of increased-risk. Effectiveness was determined by the positive predictive value (PPV) and explored in relation to 1-sensitivity and 1-specificity.

**Results:**

Complete data was available on 43470 children (87% of the survey). At the Scotland level, at least half (50%) of the children targeted were at increased-risk irrespective of the method used to target or the definition of increased-risk. There was no one method across all definitions of *increased-risk* that maximised PPV. Instead, PPV was highest when the targeting method complimented the definition of *increased-risk*. There was a higher percentage of children at *increased-risk* who were not targeted (1-sensitivity) when caries experience (rather than deprivation) was used to define *increased-risk*, irrespective of the method used for targeting. Over all three definitions of *increased-risk*, there was no one method that minimised (1-sensitivity) although this was lowest when the method and definition of *increased-risk* were complimentary. The false positive rate (1-specificity) for all methods and all definitions of *increased-risk* was consistently low (<20%), again being lowest when the method and definition of *increased-risk* were complimentary.

**Conclusion:**

Developing a method to reach all (or even the vast majority) of individuals at *increased-risk* defined by either caries experience or deprivation is difficult using a directed-population approach at a group level. There is a need for a wider debate between politicians and public health experts to decide how best to reach those most at need of intervention to improve health and reduce inequalities.

## Background

Health improvement strategies can involve whole population approaches, targeted programmes directed at individuals (high-risk approach) or at populations at greater risk (directed-population approach), or a mixture of approaches [1-6].

The high risk approach aims to prioritise interventions to those identified (often through screening) as at *increased-risk*. While often used, its limitations are well-recognised [7]. The directed-population approach normally uses epidemiological and/or social data to define the particular population subgroup [8]. It is has been shown, however, that such approaches can also present challenges, and targeting *all* (or the majority of) individuals considered at greatest need can be difficult to achieve [9-12]. This is particularly challenging when resources are limited, disease is widely dispersed, and other social, cultural and political factors have to be considered.

Notwithstanding the above, it is recognised that a combination of approaches is often the most appropriate option for strategies aimed at health improvement and reductions in health inequality. This is in keeping with the Marmot Review [13] of 2010 which introduced the concept of “proportionate universalism”, whereby to reduce the steepness of the social gradient in health, interventions must be universal but with a scale and intensity that is proportionate to the level of disadvantage faced.

Despite recent improvements in the oral health of children in Scotland, dental caries remains a highly prevalent disease with persisting inequalities. The most recent National Dental Inspection Programme (NDIP), which monitors the oral health of Scottish primary school children, found that overall, 33% of 5 year old primary 1 (P1) children had caries experience, and in the most deprived areas of Scotland, this figure rose to 49.5% [14]. In addition it has been established that the incidence of new cavities is much higher in children with caries than those free of caries, and therefore there is an imperative to prevent the development of caries in high-risk caries free children [15].

Childsmile, the national oral health improvement programme for children in Scotland aims to use a proportionate universalism approach to improve the oral health of children and reduce inequalities in dental health and in access to dental services. Within the programme, every child born in Scotland has access (from six months of age) to a programme of care within Primary Care Dental Services (PCDS), with the intensity of support tailored to the needs of individual families. This involves dietary advice and signposting to relevant local community development activity, toothbrushing demonstration and provision of supplies for home use and twice yearly application of fluoride varnish to the teeth. Additionally, supervised daily toothbrushing is provided to every 3 and 4-year old child attending nursery school (both local authority and private). In an attempt to ensure that the scale and intensity of the intervention is proportionate to the level of need, additional support is directed to children at *increased-risk* of decay through home and community support [16], and also via a nursery/school-based fluoride varnish clinical preventive programme that targets 20% of children attending priority nurseries and primary schools. This approach complies with current guidelines recommending twice yearly application of fluoride varnish for all children, increasing to four applications per year for those deemed at *increased-risk* of caries.

The method of targeting those children most in need of the additional nursery/school-based intervention is based on ranking schools within each Health Board area in Scotland according to those with the highest percentage of P1 pupils with a home postcode in the most deprived quintile of the Scottish Index of Multiple Deprivation (SIMD) (an area-based indicator of deprivation) [17]. Within each Health Board, all P1 children within the highest ranking schools are selected to receive the intervention until 20% of the P1 population has been reached, then no further schools are selected. It has been argued, however, that in some remote and rural areas, deprivation based methods, even when applied locally as opposed to nationally for Scotland, are not appropriate. Instead it has been suggested that in these areas the method of targeting should be based on caries experience (i.e. disease prevalence).

As this directed-population approach component of the overall Childsmile intervention involves a clinical approach to prevention, in some ways it has the same limitations as those of a high-risk strategy approach in that it is not directed at the underlying determinants of disease and therefore new high-risk children will be constantly emerging [8]. Thus, while it does not attempt to identify all high-risk children at an individual level, it is relevant to determine if this clinically-based approach, delivered in a school setting, reaches those at greater need.

This paper aims to use the Childsmile model to compare the effectiveness of a number of different methods for identifying individuals at *increased-risk* when a directed-population approach is taken within the usual economic, social and cultural constraints.

The aims of the study are:

(i) To establish what proportion of children targeted for intervention are actually at *increased-risk* using four different targeting methods and three different definitions of *increased-risk*

(ii) To describe how these findings vary across Health Boards within Scotland

(iii) To explore the effect of targeting at the national versus local Health Board level

(iv) To determine for each method and each definition of *increased-risk* the proportion of children screened in and screened out at a Scotland level

(v) To identify the most effective method of targeting children for this intervention

## Methods

The investigation involved a cross-sectional population sample of Scottish primary 1 school children undergoing a routine basic dental inspection as part of the National Dental Inspection Programme (NDIP).

### Study sample

Data from the Basic NDIP inspection of Primary 1 pupils attending local authority primary schools within Scotland in 2008 was used. In Scotland, all Primary 1 (age 5) and Primary 7 (age 11) children attending local authority schools are invited to receive a basic dental inspection as part of NDIP [14]. The basic inspection categorises child dental health into three categories based on the urgency of dental attention required [14]. The study recoded the categories to identify children as either with or without obvious caries experience. A further category was created for children scheduled to receive a basic inspection but who were absent or non-compliant on the day.

### Area-based deprivation

The SIMD is the official tool employed by the Scottish Government for identifying small area concentrations of multiple-deprivation [17]. First published in 2004 and updated in 2006, 2009 and 2012, the index is based on seven domains: income, employment, health, education, housing, access to services and crime. The overall SIMD is a weighted total of the seven domain scores, which allows a relative ranking of the 6,505 datazones across Scotland. Each datazone, consisting of approximately 750 households is ranked from one, being the most deprived, to 6,505, being the least deprived. This provides a measure of how deprived any datazone is in relation to other datazones in Scotland [17].

National SIMD quintiles divide the population of Scotland into five equal groupings, incorporating 20% of the population within each quintile (population weighted). For local SIMD, quintiles are calculated at a Health Board level so that 20% of the local Health Board population is captured within each quintile.

### Data Linkage

Data linkage was undertaken by Information Services Division (ISD) Scotland to match child-level data from the Basic NDIP database (dental health, school name, child home postcode) to local (Health Board) and national SIMD quintiles based on the home postcode of the child.

### Definition of at increased-risk

Three definitions of *at increased-risk* were explored. These were (i) caries experience (yes/no) - derived from the basic dental inspection (ii) child residing in a postcode within local SIMD quintile 1 (most deprived 20%) and (iii) child residing in a postcode within national SIMD quintile 1(most deprived 20%).

### Methods for targeting

The four methods tested for targeting children at *increased-risk* were based on delivering the intervention to 20% of the P1 population (calculated on the P1 population registered for Basic NDIP). Schools were ranked according to (i) prevalence of caries experience in P1 and (ii) prevalence of SIMD quintile 1 in P1, respectively. All P1 children within selected schools were targeted on a year-group basis, meaning that all children within a P1 class were offered the intervention, irrespective of their dental health status or level of deprivation, if their school had been selected. The cut-point included the 20^th^ percentile child plus the remainder of P1 children in that school.

•*Method 1*: used Basic NDIP (2008) P1 child data:– schools within the respective Health Board ranked in descending order by proportion of children with caries experience within respective schools

•*Method 2*: using local SIMD of child home postcode:– schools within the respective Health Board ranked in descending order by proportion of children with home postcodes in the most deprived local quintile (local SIMD1)

•*Method 3*: using Basic NDIP (2008) P1 child data:– schools ranked across Scotland in descending order by proportion of children with caries experience

•*Method 4*: using national SIMD of child home postcode:– schools ranked across Scotland in descending order by proportion of children with home postcodes in the most deprived quintile (national SIMD1)

### Ethical approval

This research involved secondary analysis of an existing data set. Ethical approval has been granted from the Medical Faculty Ethics Committee, Glasgow University (Project No.: FM04908) based on the overarching evaluation of the Childsmile programme. Approval to use the NDIP data was granted from the Scottish Dental Epidemiology Coordinating Committee (SDECC) (February 2010) and data linkage was approved by the Privacy Advisory Committee within Information Services Division, Scotland.

### Statistical analysis

All analyses were undertaken within IBM SPSS Statistics version 19.

The effectiveness of each method (1–4) in targeting those at *increased-risk* (three definitions) was determined by the positive predictive value (PPV). The PPV is the proportion of children who were targeted by the respective method (denominator) that were at *increased-risk* (numerator) [18]. The PPV of each of the four methods was calculated using the three definitions of *increased-risk*.

Also of interest to the Childsmile programme was to determine the

(i) (1-sensitivity) as the false negative rate: the proportion of children at *increased-risk* (true positives) who were not targeted.

(ii) (1-specificity) as the false positive rate: the proportion of children *not at increased-risk* (true negatives) that were targeted.

These were also measured for each of the four methods according to each of the three definitions of *at increased-risk* at the Scotland level only*.*

## Results

**Figure 1:**
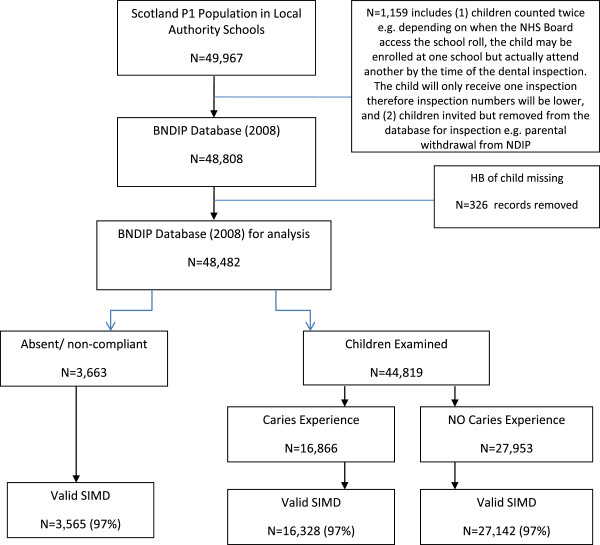
Flow diagram of analysis data.

Figure 1 presents a detailed breakdown of the sample available for analysis. Of the 49967 eligible children in Primary 1 in 2008, 43470 (87%) had complete data (caries experience and local/ national SIMD) for analysis.

### Characteristics of study sample

In 2008, 37.8% of P1 children had experience of caries. Table 1 highlights the differences in Health Board size across Scotland, with 161 P1 children registered to receive a basic dental inspection in Orkney compared to a total 11,712 in Greater Glasgow & Clyde. Mean P1 school year group size ranged from 7.1 in Western Isles to 36 in Lothian, with a national average year group size of 25 pupils. The highest proportion of P1 children with obvious caries experience is concentrated in the West of Scotland Health Boards of Greater Glasgow & Clyde, Lanarkshire, Dumfries & Galloway and Ayrshire & Arran. P1 children with a home postcode in the most deprived quintile nationally (SIMD1) are also concentrated in Greater Glasgow & Clyde (GG&C) and Lanarkshire.

**Table 1:** **Basic NDIP outcome** &**share of national SIMD1 by health board**

**Health board**	**Total children registered for BNDIP**	**Total children inspected by BNDIP**	**Mean P1 school year-group size registered for BNDIP**	**P1 children with obvious caries experience (dmft>0)**			**P1 children with no obvious caries experience (dmft>0)**			**Absent/ Non-compliant P1 children**		**National share of P1 children with home postcode in national SIMD1**	**% of national SIMD1 within Health Board**
				**N**	**% P1 children registered for BNDIP**	**% P1 children inspected by BNDIP**	**N**	**% P1 children registered for BNDIP**	**% P1 children inspected by BNDIP**	**N**	**% P1 children registered for BNDIP**		
A&A	3,449	3,438	25.3	1,326	38.4%	38.6%	2,112	61.2%	61.4%	11	0.4%	9.4%	32.4%
Bor	1,153	1,049	18.6	286	24.8%	27.3%	763	66.2%	72.7%	104	9.0%	0.7%	7.0%
D&G	1,171	1,170	13.2	475	40.6%	40.6%	695	59.4%	59.4%	1	0.1%	1.2%	12.1%
Fife	3,109	2,755	27.0	951	30.6%	34.5%	1,804	58.0%	65.5%	354	11.4%	5.4%	20.2%
FV	2,966	2,696	28.4	850	28.7%	31.5%	1,846	62.2%	68.5%	270	9.1%	4.2%	17.6%
Gram	3,602	3,254	21.7	1,051	29.2%	32.3%	2,203	61.2%	67.7%	348	9.6%	1.7%	5.3%
GG&C	11,712	10,889	33.3	4,689	40.0%	43.1%	6,200	53.0%	56.9%	823	7.0%	40.0%	39.5%
High	2,676	2,669	11.6	1,009	37.7%	37.8%	1,660	62.0%	62.2%	7	0.3%	2.9%	12.6%
Lan	6,073	5,564	27.2	2,309	38.0%	41.5%	3,255	53.6%	58.5%	509	8.4%	14.0%	26.9%
Loth	7,893	7,061	36.0	2,371	30.0%	33.6%	4,690	59.4%	66.4%	832	10.6%	12.3%	17.8%
Ork	161	140	10.7	28	17.4%	20.0%	112	69.6%	80.0%	21	13.0%	0%	0%
Shet	277	261	10.7	75	27.1%	28.7%	186	67.1%	71.3%	16	5.8%	0%	0%
Tay	3,977	3,628	24.7	1,371	34.5%	37.8%	2,257	56.8%	62.2%	349	8.7%	8.1%	23.3%
WI	263	245	7.1	75	28.5%	30.6%	170	64.7%	69.4%	18	6.8%	0%	0%
SCOT	48,482	44,819	25.0	16,866	34.8%	37.6%	27,953	57.7%	62.4%	3,663	7.5%	99.9%	

### What proportion of children targeted for intervention are at increased-risk (PPV)*?*

Table 2 presents the PPV for the three definitions of *increased-risk*, and the four targeting methods (Method 1–4) across all Health Boards in Scotland. At the Scotland level, at least half (50%) of the children targeted were at *increased-risk* irrespective of the method used to target or the definition of *increased-risk*. There was no one method across all definitions of *increased-risk* that maximised PPV. Instead, PPV was highest when the targeting method complimented the definition of *increased-risk*. This pattern was generally reflected within each of the Health Boards.

**Table 2:** **Utility of Method 1–4 – PPV- to target P1 children at increased-risk (standards 1, 2** &**3)**

**Health board**	**(1) Caries experience (dmft>0)**				**(2) Local SIMD1**				**(3) National SIMD1**			
	M1^1^	M2^2^	M3^3^	M4^4^	M1	M2	M3	M4	M1	M2	M3	M4
A&A	59.9%	51.1%	61.6%	50.3%	38.4%	70.9%	41.6%	59.0%	51.3%	74.9%	53.9%	74.7%
Bor	46.0%	36.1%	68.8%	49.2%	42.9%	72.3%	39.1%	94.3%	26.9%	28.9%	39.1%	82.9%
D&G	61.6%	48.5%	59.5%	58.8%	44.9%	68.5%	42.0%	91.2%	32.5%	44.8%	29.0%	77.9%
Fife	60.0%	53.6%	61.1%	53.7%	57.5%	69.9%	63.1%	70.5%	50.6%	64.3%	55.4%	67.4%
FV	55.9%	47.8%	60.8%	49.8%	50.0%	65.1%	54.6%	72.5%	37.7%	50.5%	42.2%	70.6%
Gram	54.2%	45.9%	57.7%	54.9%	45.9%	65.4%	56.8%	89.0%	16.0%	19.6%	21.8%	65.0%
GG&C	65.8%	59.9%	62.2%	56.2%	53.9%	72.3%	49.0%	53.3%	73.8%	88.8%	70.1%	79.9%
High	63.8%	50.0%	63.8%	55.4%	41.8%	68.1%	41.8%	88.7%	20.2%	32.4%	20.2%	83.5%
Lan	62.9%	54.7%	61.6%	52.9%	49.0%	66.8%	45.4%	62.4%	51.6%	68.8%	48.2%	68.1%
Loth	56.8%	52.7%	60.3%	54.3%	62.8%	75.4%	67.3%	79.9%	47.4%	57.9%	50.4%	71.9%
Ork	34.3%	25.0%	66.7%	N/A	25.6%	76.5%	0.0%	N/A	N/A	N/A	N/A	N/A
Shet	48.8%	32.2%	69.2%	N/A	28.6%	49.5%	25.0%	N/A	N/A	N/A	N/A	N/A
Tay	59.1%	54.0%	61.7%	51.8%	60.5%	78.2%	62.8%	74.8%	56.7%	73.9%	58.7%	71.8%
WI	50.7%	N/A^5^	68.0%	N/A	N/A	N/A	N/A	N/A	N/A	N/A	N/A	N/A
SCOTLAND	60.2%	52.8%	61.5%	54.2%	52.1%	70.5%	51.8%	63.2%	49.5%	62.2%	53.8%	75.0%

^1^Method 1 - using BNDIP (2008) P1 **child** data:– **schools** within the respective Health Board ranked in descending order by proportion of children with caries experience within respective schools.

^2^Method 2 - using local SIMD of **child** home postcode:– **schools** within the respective Health Board ranked in descending order by proportion of children with home postcodes in the most deprived local quintile (local SIMD1 2009).

^3^Method 3 - using BNDIP (2008) P1 **child** data:– **schools** ranked across Scotland in descending order by proportion of children with caries experience.

^4^Method 4 - using national SIMD of **child** home postcode:– **schools** ranked across Scotland in descending order by proportion of children with home postcodes in the most deprived quintile (national SIMD1 2009).

^5^The island Health Boards of Orkney, Shetland and Western Isles have no national SIMD1 datazones and therefore do not feature in analysis relating the methods or definitions of risk using national SIMD1. SIMD data for WI was incomplete preventing analysis by child SIMD.

### National versus local methods

#### Caries experience-based methods

**Figure 2:**
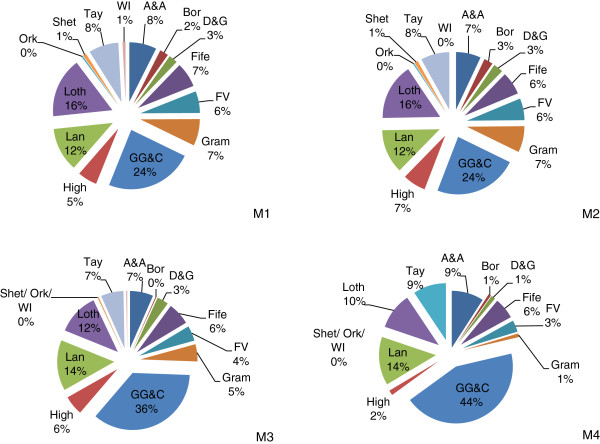
**Pie charts demonstrating proportional share of schools targeted by method by health board.** Footnote to Figure 2. M1- Method 1- Basic NDIP Local; M2- Method 2-SIMD Local; M3-Method 3. Basic NDIP-national; M4- Method 4- SIMD National.

Figure 2 demonstrates the shift in share of schools within Health Boards targeted when national methods are applied as opposed to local methods. Using caries-experience based methods (Method 1 and Method 3) when applied nationally across Scotland, as opposed to at an individual Health Board level, it is the Boards with the highest prevalence of caries that increase their share of pupils targeted. For example, GG&C increased its share from 24% to 36% and Lanarkshire from 12% to 14%.

#### Deprivation-based methods

Using deprivation based methods (Method 2 and Method 4) when SIMD is applied nationally, as opposed to locally within Health Board, it is the Boards with the highest share of national SIMD1 that increase their share of pupils targeted (Figure 2). This is predominantly Boards in the West of Scotland. For example, GG&C increased its share of pupils targeted from 24% to 44%, Lanarkshire from 12% to 14% and A&A from 7% to 9%. A corresponding decrease is seen in the remaining Boards, with no pupils targeted in the small island Boards (Orkney, Shetland, Western Isles).

#### Comparison of all four methods at a Scotland level

**Figure 3:**
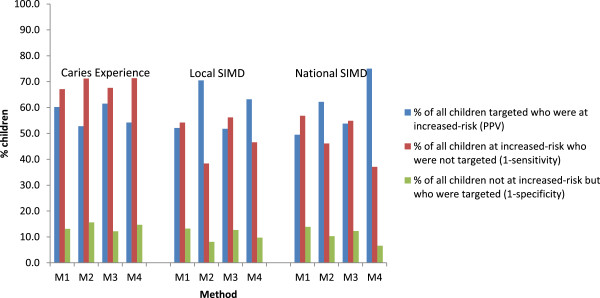
**Comparison of Scotland’s children targeted at increased-risk (defined by caries experience, SIMD local and SIMD national) by method**^**1**^**.** Footnote to Figure 3. M1- Method 1- Basic NDIP Local; M2- Method 2-SIMD Local; M3-Method 3- Basic NDIP-national; M4- Method 4- SIMD National.

Figure 3 presents the PPV, (1-sensitivity) and (1-specificity) for all four targeting methods, and three definitions of *increased-risk*, at the Scotland level. As reported earlier, the PPV was at least 50% for all targeting methods and all definitions of *increased-risk* and was highest when the targeting method and definition of *increased-risk* were complimentary. There was a higher percentage of children at *increased-risk* who were not targeted (1-sensitivity) when caries experience (rather than SIMD) was used to define *increased-risk*, irrespective of the method used for targeting. Over all three definitions of *increased-risk*, there was no one method that minimised (1-sensitivity). Instead, (1-sensitivity) was lowest when the method and definition of *increased-risk* were complimentary. The false positive rate (1-specificity) for all methods and all definitions of *increased-risk* was consistently low (<20%), again being lowest when the method and definition of *increased-risk* were complimentary.

## Discussion

While it is widely acknowledged that a directed-population approach is required to reduce the steepness of the social gradient at the population level, the implementation of this approach within the usual framework of social, economic and political constraints means that coverage of the population *at increased-risk* will rarely be 100%. Nonetheless from a public health point of view, it is important to *optimise* coverage of the intervention to the population intended, and therefore this paper aimed to compare the effectiveness of a number of different methods for targeting individuals most in need of add-itional support - when a directed-population approach is taken within an overall proportionate universalism model.

The Childsmile programme was used for illustrative purposes and the NDIP epidemiological data used to simulate the different methods. However, the principles of proportionate universalism apply to many public health intervention programmes, and this work therefore has wider application and interest.

A directed-population approach can present difficulties in identifying all of those individuals most in need of an intervention, paticularly when applied in a school (or other group) setting. Firstly, efforts must be made to prevent stigmatising individual children. This is done within Childsmile by ensuring that all P1 children within a targeted school are offered the intervention irrespective of whether they are considered at *increased-risk* or not. Secondly, all public health interventions must operate within the constraints of limited financial resources and therefore a realistic cut-off for the group to receive more intensive support must be chosen. This is achieved within Childsmile by targeting all P1 children from the highest ranking schools until 20% of the P1 population has been reached, and then no further schools are selected.

The study benefited from a large sample size utilising routine data collected on a whole population sample of Scotland’s P1 children registered to receive a basic dental inspection during 2008. The use of a widely recognised area-based indicator of deprivation (SIMD) allowed comparisons to be made across all 14 Health Boards in Scotland.

### Main Findings

The Positive Predictive Value (PPV) is a useful measure for assessing and comparing the effectiveness of different targeting methods in identifying those individuals most at need of the intervention. Sensitivity and specificity are also useful measures to complement the PPV; however it is the reciprocal of these that may be of greater interest to those responsible for the design, development and financial aspects of such an intervention programme.

Operating under the above constraints, this study found that, at a Scotland level, only around 50% of those targeted by the intervention were considered at *increased-risk*, irrespective of the method used or definition of *increased-risk*. This means that almost 50% of those targeted to receive the intervention were not considered at *increased-risk*. No single method was identified to be optimal for all three definitions of *increased-risk* investigated. For the most part, PPV was maximal when method and definition of *increased-risk* were complimentary, and patterns within Health Boards broadly reflect that of Scotland.

Furthermore, there were a substantial number of children considered at *increased-risk* who were not targeted by the methods investigated (screened out). This figure was highest when *increased-risk* was defined as caries experience. Thus, clearly the current approach is missing a significant number of *increased-risk* children.

These results highlight the difficulty in identifying a maximum number of children at *increased-risk* through a directed-population approach. Previous studies have shown that widespread distribution of caries prevalence across geographical areas, and a gradual gradient in caries severity prevents clear segmentation of *increased-risk* communities or schools [9,10] and the significant variation in areas targeted, depending on the summary measures applied [12].

Not surprisingly, this study found that SIMD methods were more effective at targeting children at *increased-risk* as defined by deprivation quintile (locally and nationally) than BNDIP methods were at targeting children with caries experience. It reflects the difference between SIMD1 as a defined quintile of the population in comparison to caries experience, which had a higher prevalence in 2008 37.6% and is diversely spread across the P1 population [14]. This finding also has parallels with the conclusions drawn by Locker *et al.*[11] that school-based targeting was more effective at identifying children from disadvantaged backgrounds; despite almost three-fifths of children overall with dental care needs not identified.

### National versus local

The current approach used in Childsmile targets 20% of the P1 population in each Health Board. Targeting nationally rather than locally would direct a greater proportion of resource to those Health Boards with a greater prevalence of disease/deprivation but would result in a less equal share of resource across the country. However, is it really fair to spread resource equally across Health Boards when both deprivation and caries experience is concentrated in greater proportions in certain parts of the country? For example, for any given Board, the local SIMD1quintile may well be less deprived (according to national SIMD) than the second most deprived quintile locally (SIMD2) for another Board area. This is particularly relevant to Greater Glasgow & Clyde which has 45.4% of the most deprived datazones (SIMD1) nationally [17]. Although this targeted intervention aims to reduce health inequalities as part of a proportionate universalism approach, by directing resources into every Health Board to reach 20% of its local population, there is the potential to widen inequalities at the national level.

It may be that resource allocation and individual objectives require to be set at Health Board level in the context of the national picture. Ultimately, this requires engagement between politicians and public health experts to determine the parameters of what is equitable.

### Caries prevalence versus SIMD measures

Whilst it has been suggested here that certain Boards benefit from the application of SIMD locally as opposed to nationally to target 20% of P1 population, some more rural Boards have argued that SIMD is a less appropriate method and advocate the use of historical Basic NDIP data in conjunction with local SIMD. Previous studies have suggested the association between deprivation and poor health is diluted in rural areas and deprived individuals with poor health are hidden by favourable population averages [19].

Although, this study found no single method offered a discernible advantage in identifying children at *increased-risk*, there are theoretical and practical reasons why we would suggest SIMD to be the preferred tool. Firstly, SIMD is a relative measure of deprivation, showing one datazone as more or less deprived in relation to another. There will always therefore be a most deprived quintile (SIMD1) and a least deprived quintile (SIMD5). In contrast, patterns of caries experience change over time and therefore offer a less stable measure for comparison year on year.

Indeed, the very premise of Childsmile School is that the oral health of children within targeted establishments will improve as a result of additional twice-yearly fluoride varnish applications. Using Basic NDIP as the tool to select schools will move schools where oral health has improved further down the ranking, with resultant withdrawal of the intervention and redirection of resource. Whilst clinical intervention has the potential to control and manage caries, without any change in the socio-economic circumstance of children attending the school, the risk has not been altered and the likelihood is that oral health status will decline on removal of the intervention. Future cohorts of children at *increased-risk* will, by nature of their circumstances, continue to materialize and require ongoing intervention. Consequently, on a further review, this school may again enter the highest ranking schools for caries prevalence and selection for targeting. Thus, methods based on Basic NDIP have the potential to create a revolving door of schools targeted and not targeted over a period of time as disease patterns change, not least because of the intervention itself. Not only is this an inefficient use of resources, but stopping and starting schools on the programme is likely to strain partnerships developed with education.

Additionally, the use of contemporaneous data utilising the 2008 BNDIP dataset to both model and test the outcome of all methods, has the potential to have artificially amplified the effectiveness of Basic NDIP methods where the predictive capacity was the best that it could be. In practice therefore it is unlikely to be able to continue to achieve the same level of result.

## Conclusions

In conclusion, this paper has shown that developing a method to reach all (or even the vast majority) of individuals at *increased-risk* defined by either caries experience or deprivation is difficult using a directed-population approach at a group level. For the Childsmile model, however, it was determined that a targeting method based on deprivation (in this case SIMD) was most appropriate, primarily due to relative ranking and stability of the SIMD in comparison to caries prevalence. For the purposes of this study the 20% cut-point for targeting was applied across methods and Health Board areas as used currently in the national programme. However, it may be that some local variation is required to reflect a greater need (as demonstrated by share of national SIMD and caries prevalence) in certain geographic areas of Scotland. The issues around the choice of local or national-based approaches have been raised, and the need for a wider debate around these issues discussed.

Furthermore, the results suggest the need to investigate the utility of this component of Childsmile, over-and-above that available via the universal elements of the programme. A randomised controlled trial is being undertaken to investigate this further.

Whilst supporting the concept of proportionate universalism, this study shows the challenges associated with attempting to provide more intensive support to those at *increased-risk* of disease and of achieving attendant reductions in health inequality, particularly when the intervention is clinically based and does not address the underlying determinants of inequality. It provides the background from which to engage politicians and public health experts in an open debate on how best to reach those most in need and thus begin to address the issue of inequalities locally and globally.

## Abbreviations

NDIP: National dental inspection programme; PCDS: Primary care dental services; SIMD: Scottish index of multiple deprivation; ISD: Information services division; P1: Primary 1; P7: Primary 7; PPV: Positive predictive value.

## Competing interests

The authors declare that they have no competing interests.

## Authors’ contributions

LB and LM conceived of the study. LB, LM and AS were involved in the design of the study. LB and AS performed the statistical analysis of the data. All authors were involved in the interpretation of results, drafting the manuscript and reading and approval of the final manuscript.

## Pre-publication history

The pre-publication history for this paper can be accessed here:

http://www.biomedcentral.com/1471-2458/13/778/prepub
